# The effects of participation level on recidivism: a study of drug treatment courts using propensity score matching

**DOI:** 10.1186/1747-597X-9-40

**Published:** 2014-09-24

**Authors:** Elizabeth J Gifford, Lindsey M Eldred, Sabrina A McCutchan, Frank A Sloan

**Affiliations:** Center for Child and Family Policy, Duke University, Box 90545, 302 Towerview Drive, Durham, NC 27708 USA; Department of Economics, Duke University, 213 Social Sciences Building, Box 90097, Durham, NC 27708 USA

## Abstract

**Background:**

Empirical evidence has suggested that drug treatment courts (DTCs) reduce re-arrest rates. However, DTC program completion rates are low and little is known about the effectiveness of lower levels of program participation.

**Objectives:**

We examined how DTC program referral, enrollment without completion, and completion, affected re-arrest rates during a two-year follow-up.

**Research design:**

We used statewide North Carolina data from criminal courts merged with DTC data. Propensity score matching was used to select comparison groups based on demographic characteristics, criminal histories, and drug of choice (when available). Average treatment effects on the treated were computed.

**Measures:**

DTC participation levels included referral without enrollment, (n = 2,174), enrollment without completion (n = 954), and completion (n = 747). Recidivism measured as re-arrest on a substance-related charge, on a violent offense charge not involving an allegation of substance abuse, and on any charge (excluding infractions) was examined by felony and misdemeanor status during a two-year follow-up period.

**Results:**

Re-arrest rates were high, 53–76 percent. In general, re-arrest rates were similar for individuals who were referred but who did not enroll and a matched comparison group consisting of individuals who were not referred. In contrast, enrollees who did not complete had lower re-arrest rates than a matched group of individuals who were referred but did not enroll, for arrests on any charge, on any felony charge, and on substance-related charges (felonies and misdemeanors). Finally, relative to persons who enrolled but did not complete, those who completed had lower re-arrest rates on any charge, any felony charge, any misdemeanor charge, any substance-related charge, any substance-related misdemeanor or felony charge, and any violent felony charge.

**Conclusions:**

Enrolling in a DTC, even without completing, reduced re-arrest rates. Given the generally low DTC completion rate, this finding implies that only examining effects of completion underestimates the benefits of DTC programs.

## Background

The link between substance use and crime is longstanding and well documented. Among state prisoners, estimates of drug use at the time of a criminal offense are as high as 30 percent and more than half had used drugs in the month prior to arrest [1]. The criminal justice system has attempted to confront this problem in part by creating specialized drug treatment courts (DTCs) that provide a structured treatment program with court oversight in lieu of or in addition to traditional penalties. Since the first drug court opened in Dade County Florida in 1989, state legislatures and individual courts have created specific statutory provisions authorizing the creation of and providing funding for DTCs, which are defined in this study as adult drug treatment courts or specialized courts for treating adults with an arrest on a Driving While Intoxicated (DWI) charge, i.e., DWI courts. As of 2012, there were 2,700 DTCs in the United States and 25 in North Carolina [2].

There have been numerous evaluations of the effectiveness of DTCs in reducing recidivism and substance use [3–7]. Overall, most research has indicated that DTCs lower re-arrest, re-conviction, and substance use rates [6, 8–12]. However, results have been mixed regarding the effectiveness of DTCs [7, 10]. For example, results of a meta-analysis of 55 evaluations on the effect of such courts on recidivism suggested that drug offenders who participated in a DTC program were less likely to re-offend; but, this relationship was weaker when more robust methods were used [7]. Similarly, a recent review of 154 independent evaluations of DTC programs reported that evaluations based on more rigorous designs found smaller effect sizes [10]. Researchers skeptical of positive findings have suggested that DTCs may selectively serve persons with the lowest level of addiction [13–15], that studies used inadequate comparison groups [16, 17], or that studies were based on a biased definition of the treated group [17]. Past studies using randomized controlled trials (RCTs), generally considered to be the gold standard, have found that DTCs were less effective than those based on observational data [9, 18]. However, RCTs may not fully capture details of DTC implementation, or DTCs in actual practice may tend to operate differently from those willing to participate in RCTs. Also, less successful DTCs may be less willing to participate in RCTs.

Evaluations of DTCs based on observational data frequently have involved matched comparison groups and have used propensity score matching in particular [19–21]. Given the paucity of attributes of offenders available in such data, even after matching on available attributes, the possibility remains that there were differences between DTC and comparison group participants on important attributes that may have independent effects on outcomes.

Using longitudinal statewide data from North Carolina, this study examined whether being referred to, enrolling in, and/or completing a DTC program changed behavior for a criminal offender with a substance use problem. Our study offered several advantages over previous studies of DTCs. First, we examined differences of re-arrest rates between three groups of DTC participants, those who (1) were referred but did not enroll, (2) enrolled but did not complete and (3) completed. We hypothesized that, holding other factors constant, higher levels of participation in a DTC program would be associated with reduced recidivism. That is, completing a DTC program would be more effective than enrolling in but not completing the program and enrolling in the program would be more effective than just being referred. Simply being referred to or enrolling in a DTC program may prevent future criminal activity by exposing the prosecuted or convicted person to available community resources for individuals with addictions. This is particularly relevant since far greater numbers of persons are referred to DTCs than who actually enroll, and many more persons enroll without completing [22]. Yet, existing studies have tended to compare those who completed a DTC program (or graduates) with those who dropped out of a program [11, 23, 24]. Therefore, we questioned whether the resources that are spent on persons who are referred to but who do not enroll or who enroll but do not complete are entirely wasted or if there is at least a partial benefit.

Second, compared to previous studies, we enhanced the generalizability of findings by including data from 19 DTCs in a populous state, North Carolina (NC), during 2005–2012. Third, our analysis used linked administrative data rather than self-reports from sample surveys. Fourth, we examined effects of DTC programs on recidivism for all types of crimes, not only those directly related to substance use or trade in illicit substances.

## Background on drug treatment courts

DTCs aim to reduce the incidence of drug and alcohol-related crimes (N.C. Gen. Stat. § 7A-791). Judges, prosecutors, defense attorneys, and probation officers may refer participants to a DTC. Enrollment in DTCs in North Carolina is voluntary, and DTC referral occurs post plea. These programs have three phases, even though attendance and treatment requirements for each phase vary across courts. Further, while each individual court has the discretion to determine which type of sanctions and rewards to issue, the types of rewards and sanctions used by the courts are consistent among all DTCs in the state. For example, the most common sanctions in fiscal year 2008–9 were jail for 24–48 hours (35%), individualized sanction (16%), and community service (6%) [25].

Multiple factors may encourage or discourage program completion. One factor that may encourage program completion is suspended sentences whereby offenders who complete the program do not serve their original sentence. In North Carolina, if a sentence is suspended pending successful completion of a DTC, failure to complete the DTC program will result in reinstatement of the original sentence. On the other hand, strict court rules may discourage completion. For example, participants who test positive for drugs while in the program, or who fail to attend court as scheduled may be terminated from the program at any time. Because participation is voluntary, participants may choose to discontinue their participation at any time. Completion of a DTC program may take a year or more. Among those that we observed to have completed the program, the mean length of time to completion was 435 days (standard deviation, 172 days).

The most common type of DTC is the adult drug treatment court. In these, referrals are not limited to persons convicted of a substance-related crime. The general eligibility requirements are the same among all DTCs in North Carolina. To be considered for referral, individuals need to be: addicted to a chemical substance; willing to volunteer for the drug treatment court program, and eligible under the structured sentencing system for a community or intermediate punishment as an alternative to active prison time. However, each county drug program may impose additional eligibility criteria.

Another type of DTC, the DWI court, focuses on individuals arrested for DWIs. In North Carolina, DWI courts focus on persons convicted of a DWI who have been sentenced to supervised probation and who were found to have had at least one of the following aggravating factors: (1) driving while license revoked as a result of DWI, (2) causing injury to another person as a result of DWI, or (3) DWI with a child under the age of 16 in the vehicle [26].

## Methods

### Data

This study relied on two administrative databases from the NC Administrative Office of the Courts. The first was the Drug Treatment Court Management Information System (DTC-MIS) database, which tracks individuals referred, enrolled, and graduated from the 19 state-funded drug treatment court programs. The second database, the Automated Criminal Infraction System (ACIS), contained information on all arrests for felonies, misdemeanors, infractions, and traffic offenses in the state from 2005 through 2012. Infractions, which in North Carolina are non-criminal offenses not punishable with jail time (e.g., use of improper equipment, failure to wear a seatbelt), were excluded from the analyses.

Information from the ACIS was merged with the DTC-MIS data to document an individual’s court involvement. The DTC record was merged with the most recent ACIS record prior to DTC referral. In total, 3,875 of 5,257 DTC-MIS referrals to DTC and ACIS records were merged (73.7%). Failure to merge may reflect recording errors, e.g., name changes, misspelled names, or errors in data entry of birthdates.

### Analysis sample

The observational unit was a person-index arrest. For persons never referred to a DTC, index arrests were defined as the first criminal arrest in a calendar year. An individual could thus have up to four index arrests in the sample, one for each year 2007–2010. For persons referred to a DTC, the index arrest was defined as the arrest corresponding to referral. The analyses examined three types of involvement with the courts: being referred but not enrolling in a DTC (n = 2,174), enrolling in a DTC but not completing the program (n = 954), and completing the DTC program (n = 747). The presence of a referral date in the DTC data identified individuals who were referred. Enrollment was determined by the presence of a recorded admission date, treatment phase-one entry date, or a discharge date. Program completion was determined by satisfying one of the following conditions: (1) the person completed Phase 3 treatment, the final phase of active treatment, (2) the person entered Phase 4 treatment, aftercare, or another post-completion program, or (3) the discharge record indicated the person completed the program.

### Measures

Re-arrest during a two-year follow-up period was the outcome measure. Binary variables indicated if the person was arrested for: (1) a substance-related charge; (2) a charge involving a violent offense excluding those that co-occurred with a substance-related crime, which was infrequent—only 2.7 percent of violent offenses co-occurred with a substance-related crime; and (3) on any charge, excluding infractions.

Offenses associated with the index offense accounted for the fact that individuals often incurred several charges on the same day. We developed a hierarchy based on the assumption that substance abuse charges were most likely to directly lead to a referral to a DTC. In descending order, the hierarchy consisted of charges for the following offenses: both a drug and a DWI; a DWI only; drug only; violent crime; alcohol (non-DWI); traffic; and other. We further subdivided these categories into arrests on felony or misdemeanor charges, with felony given a higher priority. For example, if a person had both a drug felony and a violent misdemeanor charge for the index arrest, the index arrest was classified as a drug felony. This same hierarchy was used to classify arrests for offenses that occurred in the two-year look-back period.

Drug of choice was classified into mutually exclusive variables representing: alcohol; cocaine-powder; crack; heroin; other narcotics; other or no drug of choice; and a binary variable for missing values. This information was not available for individuals who were not referred to a DTC program.

### Statistical analysis

Our statistical approach compared differences in criminal recidivism across groups using propensity score matching (PSM). PSM is a statistical approach commonly used in observational studies where it is not feasible to randomly assign individuals to a treatment condition [27]. Randomization has become the standard for determining statistical causation, in part because it balances both observed and unobserved differences between treatment and control groups [28]. PSM has been used to balance characteristics between treatment and comparison groups on observable characteristics [29]. To the extent that unobserved characteristics were correlated with the observed variables, PSM helped to control for such heterogeneity.

The three treatment groups considered in this study were those who: (1) were referred but did not enroll; (2) enrolled but did not complete; and (3) completed. To help mitigate issues of heterogeneity of characteristics across groups, the comparison group for each treatment group was drawn from individuals who were most likely to match the treatment group. Comparison groups were drawn from individuals with the next lowest level of participation. Specifically, to compare outcomes with those who were referred but did not enroll with individuals who were not referred, a matched comparison sample was drawn from the 19 counties that had a DTC program. This was done in an effort to balance county characteristics that could have affected re-arrest rates such as resources dedicated toward policing. For the analysis of the effects of enrollment, the comparison group consisted of persons who were referred but did not enroll. Finally, to analyze the effects of completion, the comparison group consisted of persons who were enrolled but did not complete the program.

The propensity score for the probability of referral to a DTC was modeled using a logistic regression. One-to-one nearest neighbor matching with a caliper of 0.01 was done using psmatch2 in Stata version 11 [30, 31]. Treatment and comparison groups were matched on the following characteristics: age, gender, and race (black, Hispanic, other, white (omitted)), offense committed at arrest, year of offense, offense of prior arrests in a two year window, drug of choice (when available), and whether a DTC existed in the county where the arrest occurred.

To assess the quality of the match, we calculated standardized differences of each covariate before and after matching. A standardized difference compares mean differences in units based on the pooled (treatment plus comparison) standard deviation [32]. The standardized difference for binary variables is:


where  and  indicate the prevalence of the dichotomous variable in the treatment group and the comparison group, respectively. Then, for each outcome we calculated the average treatment on the treated effect (ATT) and gauged statistical significance using t-tests.

## Results

### Matching

Before matching, persons referred to a DTC program differed from the larger population of individuals who had been arrested but who were not referred, as indicated by many standardized differences greater than 10 percent (Table 1). After matching, standardized differences were all less than 10 percent. Prior to matching, differences between individuals who enrolled but did not complete and those who were referred but did not enroll were smaller than the differences between those who were referred but did not enroll and those who were not referred.

**Table 1:** **Descriptive statistics: means and standardized differences before and after matching**

	Referred				Enrolled				Completed			
	Referred & did not enroll (T) vs. not referred (C)				Enrolled & did not complete (T) vs. referred & did not enroll (C)				Completed (T) vs. enrolled & did not complete (C)			
	Without matching			With matching	Without matching			With matching	Without matching			With matching
	(N _t_ = 2,174; N _c_ = 1,561,225)			(N _t_ = N _c_ = 1,989)	(N _t_ = 954 N _c_ = 2,174)			(N _t_ = N _c_ = 887)	(N _t_ = 747 N _c_ = 954)			(N _t_ = N _c_ = 600)
	T (%)	C (%)	StDif	StDif	T (%)	C (%)	StDif	StDif	T (%)	C (%)	StDif	StDif
**Demographics**												
Age^ŧ^	35.6	35.1	4.7	4.3	33.7	35.6	−19.9	0.2	36.8	33.7	32.5	7.1
Female	34.1	32.1	4.1	8.0	35.2	34.1	2.4	−2.1	32.4	35.2	−6.0	−4.2
Black	44.2	36.8	15.1	−1.9	40.7	44.2	−7.1	1.6	43.8	40.7	6.3	4.7
Hispanic	0.8	12.3	−47.6	0.0	0.9	0.8	1.2	−1.1	2.1	0.9	9.7	1.3
Other Race	0.6	3.2	−18.8	5.1	0.6	0.6	−0.2	1.6	0.5	0.6	−1.2	0.0
**Index Arrests**												
Drug & DWI	0.6	0.2	7.2	−3.9	0.7	0.6	1.7	6.0	1.6	0.7	8.1	−7.3
DWI Felony	0.3	0.0	6.4	0.0	.	0.3	−7.4	−4.7	0.3	0.0	7.3	.
DWI Mis.	8.8	4.4	18.0	0.0	13.2	8.8	14.0	4.0	28.6	13.2	38.6	0.0
Drug Felony	41.0	1.8	108.9	3.2	35.3	41.0	−11.8	−5.1	38.6	35.3	6.7	4.1
Drug Mis.	8.0	3.0	22.3	−5.2	6.6	8.0	−5.4	0.4	5.1	6.6	−6.5	−3.4
Violent Felony	1.4	0.5	8.6	0.4	0.7	1.4	−6.3	2.7	0.5	0.7	−2.5	−4.1
Violent Mis.	2.8	4.1	−7.2	5.4	2.7	2.8	−0.2	0.0	0.7	2.7	−16.0	4.1
Alcohol Felony or Mis.	0.8	1.8	−8.6	−2.1	0.9	0.8	1.2	3.0	0.5	0.9	−4.8	−1.9
Traffic	7.9	72.8	−176.4	1.7	5.6	7.9	−9.2	−4.2	5.0	5.6	−2.7	−2.1
Other Felony	17.8	2.3	53.4	−1.9	21.8	17.8	10.1	0.3	12.4	21.8	−25.0	1.4
Other Mis.	10.6	9.2	4.7	−0.2	12.4	10.6	5.5	3.6	6.7	12.4	−19.4	−1.2
**Arrest History**												
Drug & DWI	1.0	0.2	10.6	−1.5	1.7	1.0	5.8	2.0	1.5	1.7	−1.6	−3.8
DWI Felony	0.2	0.0	5.4	1.8	.	0.2	−6.1	−6.7	0.1	0.0	5.2	.
DWI Mis.	11.1	3.0	32.2	−5.6	13.4	11.1	7.0	1.3	18.9	13.4	14.9	−3.7
Drug Felony	22.0	2.2	63.9	−1.9	17.4	22.0	−11.5	2.7	10.8	17.4	−18.9	−3.4
Drug Mis.	17.1	3.0	48.3	0.9	17.7	17.1	1.6	−2.3	13.1	17.7	−12.7	−0.5
Violent Felony	1.0	0.5	6.5	2.7	1.3	1.0	2.3	−3.1	.	1.3	−16.0	−16.4
Violent Mis.	6.0	3.1	13.9	3.4	7.7	6.0	6.6	0.9	3.2	7.7	−19.7	3.5
Alcohol Felony or Mis.	2.0	1.1	7.3	3.7	1.7	2.0	−2.6	−0.9	2.3	1.7	4.3	1.2
Traffic	16.2	28.0	−28.7	−0.3	17.3	16.2	2.8	−3.5	22.0	17.3	11.7	0.0
Other Felony	5.1	0.8	25.6	0.9	5.1	5.1	0.1	3.6	3.5	5.1	−8.2	7.1
Other Mis.	3.4	2.3	6.4	−1.6	3.9	3.4	2.5	−2.3	2.8	3.9	−5.9	0.0
None	14.8	55.8	−95.0	2.6	12.9	14.8	−5.6	2.0	21.8	12.9	23.7	6.1
**Drug of Choice**												
Alcohol	11.4	Data Not Available			19.6	11.4	22.9	38.1	35.1	19.6	35.2	3.5
Cocaine Powder	3.4				8.6	3.4	22.0	0.8	7.6	8.6	−3.5	1.2
Crack	14.7				30.5	14.7	38.5	−1.7	22.0	30.5	−19.5	2.3
Marijuana	7.0				14.4	7.0	24.0	0.7	15.1	14.4	2.2	−0.4
Heroin	5.7				11.0	5.7	19.1	−0.7	6.8	11.0	−14.7	2.4
Other Narcotics	5.0				8.5	5.0	14.1	−2.4	7.9	8.5	−2.2	0.0
“None” or “Other”	1.3				1.8	1.3	3.6	−1.6	1.2	1.8	−4.8	0.0
Missing Value	51.5				5.7	51.5	−117.8	−36.7	4.3	5.7	−6.3	−14.9
Index Year^ŧ^	2008.3	2008.6	−21.3	1.9	2008.2	2008.3	−10.4	−4.1	2008.2	2008.2	1.5	−4.8
DTC in County	96.2	100.0	−28.0	.	96.4	96.2	1.1	0.0	97.9	96.4	8.5	3.3

Note: Mis. = misdemeanor; T = treatment; C = comparison; StDif = standardized difference.

^ŧ^Mean (all other numbers are percent).

Among individuals referred, we also matched on drug of choice. A higher percentage of the referred sample listed alcohol, cocaine, crack, marijuana, heroin, and other narcotics relative to those who enrolled but did not complete. Relative to those who enrolled but did not complete, a much higher percentage of those who were referred but did not enroll had missing information. After matching, all standardized differences except for two categories related to drug of choice, alcohol and missing, were under 10 percent.

### The effect of participation in a DTC program on two-year recidivism rates

In the matched comparison sample, nearly three-quarters of individuals who were referred to a DTC program but who did not enroll were re-arrested for an offense (other than an infraction) during the two-year follow-up period (Table 2). The only statistically significant difference between those who were referred but did not enroll and those who were not referred was for re-arrest for a violent offense—which was 1.7 percentage points higher for individuals who were referred but did not enroll (*t =* 2.02, *d.f. =* 3,976, *p =* 0.043).

**Table 2:** **Effects of drug treatment court participation on two-year recidivism**

	Referred			Enrolled			Completed		
	Referred but did not enroll (T) vs. not-referred (C)			Enrolled but not complete (T) vs. referred but did not enroll (C)			Completed DTC (T) vs. enrolled but did not complete (C)		
	(N _t_ = N _c_ = 1,989)			(N _t_ = N _c_ = 887)			(N _t_ = N _c_ = 600)		
	T (%)	C (%)	ATT	T (%)	C (%)	ATT	T (%)	C (%)	ATT
Re-arrest	73.2	71.2	2.0	66.7	75.9	−9.1**	53.3	64.5	−11.2**
Felony	20.8	19.8	1.0	14.0	22.9	−8.9**	6.3	11.8	−5.5**
Misdemeanor	52.4	51.4	1.0	52.8	53.0	−0.2	47.0	52.7	−5.7*
Re-arrest for a substance-related offense	31.9	31.0	0.9	24.4	35.1	−10.7**	17.3	23.0	−5.7*
Felony	13.8	13.5	0.3	9.4	15.2	−5.9**	4.2	8.3	−4.2**
Misdemeanor	18.0	17.4	0.6	15.0	19.8	−4.8**	13.2	14.7	−1.5
Re-arrest for a violent offense	8.5	6.8	1.7*	7.1	8.1	−1.0	4.0	6.5	−2.5
Felony	2.1	1.6	0.5	1.6	2.1	−0.6	0.2	1.5	−1.3*
Misdemeanor	6.5	5.2	1.3	5.5	6.0	−0.5	3.8	5.0	−1.2

Note: **p < 0.01, *p < 0.05; T = treatment; C = comparison; ATT = Average treatment effect on the treated.

ATTs were calculated using t-tests following propensity score matching.

Those who enrolled but did not complete were less likely to be re-arrested for any offense (other than an infraction) during the two-year follow-up than the matched sample of individuals who were referred but did not enroll. Two-thirds of individuals who enrolled but did not complete were re-arrested within two years relative to three-quarters of persons referred but who never enrolled (*t =* 4.27, *d.f*. = 1,772, *p <* 0.001). Nearly all of this reduction was attributable to a reduction in arrests on felony charges, (*t =* 4.87, *d.f. =* 1,772, *p <* 0.001). The ATT for substance-related arrests was −10.7 percentage points (*t =* 4.97, *d.f =* 1,772, *p <* 0.001), indicating that those who enrolled but did not complete were statistically significantly less likely to be re-arrested for a substance-related offense than individuals who were referred but who did not enroll. These groups were not statistically significantly different on re-arrest rates for violent offenses.

Program completion was also effective in reducing recidivism as measured by any re-arrest, and the difference in the matched sample between completion and enrolling without completion was 11.2 percentage points (*t =* 3.95, *d.f. =* 1,198, *p <* 0.001). Even so, half of those who completed a DTC program were re-arrested on some charge, not including infractions, within two years. Those who completed a DTC program were also less likely to be re-arrested for a substance-related offense than the matched comparison sample of individuals who enrolled in a DTC program but did not complete (*t =* 2.45, *d.f. =* 1,198 , *p =* 0.014). Nearly all of this difference was attributable to a reduction in substance-related felony arrests in the group who completed a DTC program. While rare in both groups, a smaller percentage of those who completed the DTC program were re-arrested for a violent felony offense than the matched comparison group of individuals who enrolled but did not complete; the ATT for this comparison was −1.3 percent (*t =* 2.55, *d.f. =* 1,198, *p =* 0.011).

## Discussion

We found substantial reductions in rates of re-arrest among both persons who enrolled in a DTC program but did not complete and those who completed a DTC program. In contrast, just referring a person to a DTC did not reduce recidivism rates for any of the outcomes examined in this study. Importantly, while completion was beneficial in terms of reducing re-arrest, even enrolling without completion led to a substantial improvement in re-arrest rates during the two years following the date of the index arrest. Most prior studies focused solely on differences between graduates and dropouts. Enrollment in a treatment court program substantially decreased recidivism rates for many types of crime, particularly for substance-related crimes.

Many DTC programs across the country do not accept persons convicted of a violent crime. Nevertheless, we documented a 1.3 percentage point decrease in re-arrest for violent felony offenses when comparing those who completed a DTC program with those who enrolled but did not complete, which implies that reduced violent crime was a positive externality of a completed substance abuse treatment program. Substance use is a risk factor for serious violent crimes such as domestic violence, child abuse, assault, and murder [33]. Documenting a decrease in violent crime in response to DTC completion may be particularly appealing to the public, which may be more willing to financially support programs that prevent serious crimes [34].

Despite the positive impact of treatment courts on recidivism, participation in these courts was low. Fewer than 5,000 individuals were referred to a treatment court program over a four-year window for the entire state as contrasted with over a quarter million people who were arrested for a substance-related offense during that same time period. In our sample of participants, only 44 percent of referred persons enrolled and only 19 percent completed. This was lower than completion rates of 27–66 percent documented in other studies [35]. Our study also found that, contrary to our hypothesis, referral alone had no significant impact on reducing recidivism for any type of crime. To maximize the societal impact of treatment courts, then, efforts must focus on improving enrollment and completion rates rather than on increasing referrals.

Our study had several strengths. First, the analysis applied to all state-funded DTCs, not just to individual treatment courts or to courts that agreed to participate in a research study. Second, administrative data offer advantages over survey data based on self-reports. Not only are the samples generally much larger, but administrative data are not subject to recall bias as are self-reported data on arrests and disposition of the arrests. Third, our analysis considered effects of DTCs separately for various stages in the completion process. We found no effects for just being referred, indicating that some treatment is a prerequisite for program effectiveness.

We acknowledge several limitations. First, we relied exclusively on administrative data, which only included a few participant characteristics. This omission potentially adversely affected the quality of the matches between treatment and comparison groups. There may be unobserved differences in such factors as financial circumstances, social support, motivation to change behavior, and underlying comorbid mental health or addiction disorders. Such differences may have persisted across our study groups based on DTC participation levels—for example those who were referred but did not enroll may have been less likely than referred individuals who enrolled to believe that treatment services would benefit them. Similarly those who completed a DTC program may have had better support networks to facilitate completion relative to individuals who enrolled but did not complete. However, complete, reliable, longitudinal information on court participation is rarely available to researchers. Although detailed surveys of alcohol and illicit drug use have been conducted, these surveys lack questions about arrests, arrest disposition, and respondent involvement in DTC programs.

Second, we analyzed DTCs in North Carolina as a group rather than explicitly account for differences in organizational characteristics. While several studies have focused on individual factors such as gender and employment that affect completion [36–38], few studies have examined organizational factors related to completion rates [35]. Such organizational factors include the judge referral and supervision process, level of supervision provided, and level of sanctions imposed for not complying with program requirements [35]. Courts vary in the amount of leverage they have over participants—the consequences faced by participants who do not meet program requirements, and the rewards granted to participants for progressing through the program, vary from court to court [39].

A third limitation involved the use of official state records to document recidivism. This measure may underreport criminal recidivism rates as individuals who engage in illegal activities are often not arrested [40, 41]. Relatedly, individuals who participated in a DTC program may have experienced additional monitoring by the legal system and may have been more likely to be caught. This could suggest that our results underestimate the true reduction in recidivism.

Fourth, results from one state may not generalize to the U.S. as a whole. While many DTCs in the U.S. follow the model set out by the National Drug Court Institute and the National Association of Drug Court Professionals, there are important variations in implementation, e.g., in the sanctions imposed on those who fail to complete the program [42, 43].

These findings are relevant in light of ongoing policy debates about specialty court funding [44]. The societal costs of substance use treatment have gained considerable attention through the war on drugs and the associated increase in the prison population. A recent study of a California program aimed at diverting drug offenders into treatment suggests an average savings of $2,317 per offender over a 30-month post-conviction time span [45]. Most savings were attributed to reduced levels of incarceration. The relatively large numbers of individuals who enrolled in these courts and received some treatment, but did not complete the program, may mitigate these savings. Many DTCs have policies that require individuals who do not complete treatment to serve their original sentences, thus, costing the state for the DTC expenses as well as incarceration expenses [4, 46]. Future research should focus on factors that improve enrollment and completion rates so that the DTC model may be more efficiently implemented.
